# Advancing sustainable thermal power plants: A thermodynamic-based condition monitoring model for control valves in gas turbines under thermal fluctuations

**DOI:** 10.1038/s41598-026-47688-z

**Published:** 2026-04-10

**Authors:** Momeni Amir Hossein, Akilu Yunusa-Kaltungo, Noha A. Mostafa

**Affiliations:** 1 Mechanical Engineering Department, Shiraz University, Shiraz, 7193616548 Iran; 2 Engineering Department, Mapna Power Service Company, Tehran, 1919613871 Iran; 3 Department of Mechanical and Aerospace Engineering, Faculty of Science and Engineering (FSE), School of Engineering, The University of Manchester, Manchester, UK; 4 Industrial Engineering Department, Faculty of Science and Engineering, Zagazig University, Zagazig, 44519 Egypt; 5 Mechanical Engineering Department, Faculty of Science and Engineering, The British University in Egypt, Sherouk, 11837 Egypt

**Keywords:** Natural gas, Combustion process, Control valve, Statistical condition monitoring, Thermal power plant, Energy science and technology, Engineering

## Abstract

Precise monitoring of control valve performance is essential for regulating combustion outlet temperature, minimising nitrogen oxides emissions, and maintaining stable combustion in natural gas turbines. This study examines the temperature variations of a natural gas flow control valve, taking into account changes in valve stem position and cross-sectional area within a gas turbine power plant. A unique model is introduced to regulate valve area variation and assess performance based on the correlation between upstream and downstream temperatures. The model elucidates the impact of fluctuations in the valve cross-sectional area on the downstream natural gas temperature, taking into account upstream temperature variations between 285.15 K and 305.15 K. The Joule–Thomson coefficient is obtained from thermodynamic equations by the AGA8-92DC numerical approach. The gas composition, inversion characteristics, upstream and downstream temperature differentials, and the temperature gradient relative to the valve cross-sectional area were computed for two samples of natural gas and pure methane. The results demonstrate the differences in upstream and downstream temperatures at two pressure levels (2 MPa and 5.4 MPa). The comprehension of the temperature differential between the upstream and downstream areas can act as a crucial metric for condition monitoring and assessing the efficacy of fuel injection in control valves.

## Introduction

**Fig. 1:**
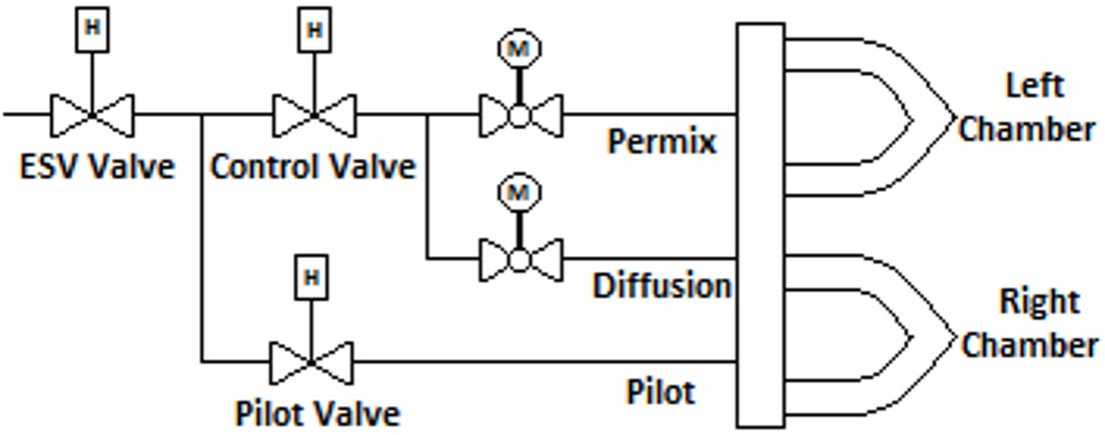
Schematic representation of the pipeline combustion process of a gas turbine power plant.

**Fig. 2:**
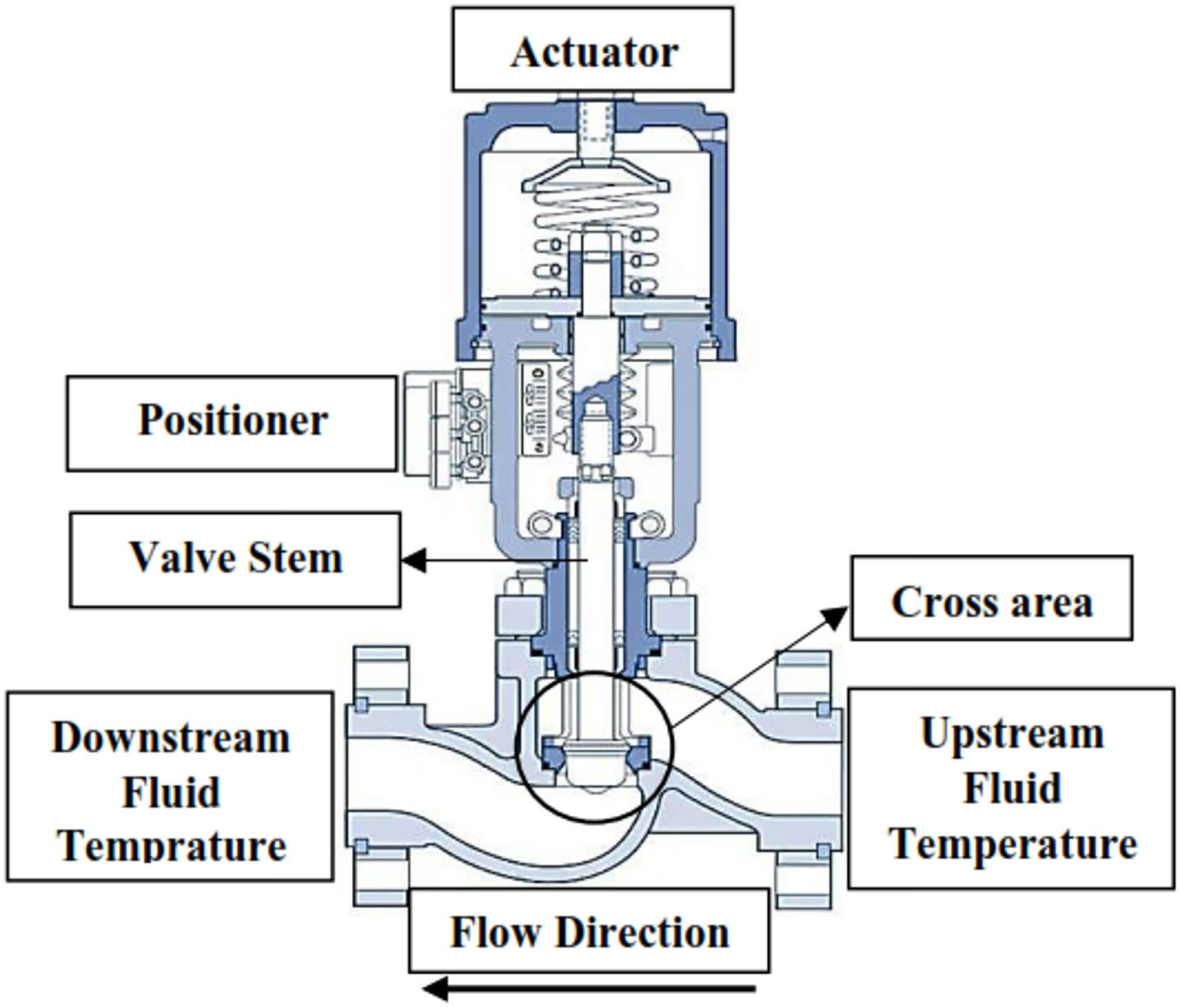
Schematic representation of a hydraulic natural gas control valve.

Gas turbines are fundamental to various power generation processes; thus, monitoring the control valves during combustion is essential for proper combustion management^1,2^. Ensuring the optimal functionality of this valve not only prolongs the lifespan of components subjected to combustion byproducts but also offers substantial advantages in regulating nitrogen oxides (NOx) emissions, hence enhancing sustainability^3–5^. Furthermore, it enhances the precision and reliability of the combustion cycle^6^. This work presents a novel technological and statistical model to examine the fluctuation in the cross-sectional area of natural gas flow resulting from alterations in the control valve stem position. This change results in a quantifiable temperature differential between the upstream and the downstream sides of the control valve. Monitoring this temperature differential can act as a crucial indicator for condition assessment, facilitating the evaluation of the fuel injection control valve’s performance^7^. Figure 1 illustrates a schematic representation of the piping system in a natural gas power plant gas turbine. Combustion within the chambers transpires in both premixed and diffusion phases. Monitoring the control valve’s performance during the ongoing combustion process is essential for accuracy and dependability. Figure 2 illustrates a schematic of a hydraulic natural gas control valve, indicating the fluid temperatures upstream and downstream.

Numerous studies have examined the performance of control valves at varying temperatures. Hossain et al.^8^ investigated the behaviour of two direct drive valves actuated by a rotary solenoid and stepper motor, examining the influence of friction and temperature on the response of valves; they also evaluated the impact of inlet control valves on the total performance of the limaçon expander. This work presents a technical model to assess the impact of natural gas flow through the gas turbine control valve. The precision of the control valve’s operation is assessed by measuring the temperature differential between the upstream and downstream flow, in correlation with the valve’s opening position. The examination of vortex shedding effects on combustion burners, caused by the positioning of instruments for downstream temperature measurements, imposes constraints on the implementation of experimental testing. These limitations must be considered in subsequent studies. As the cross-sectional area of the control valve fluctuates, the downstream pressure stays rather stable, with deviations confined to a few millibars^9,10^. Nonetheless, the flow rate and temperature of natural gas will fluctuate correspondingly. This technical model is applicable for gas turbine performance analysers and control system designers.

In gas turbine fuel systems, the control valve operates under throttling conditions, where a reduction in pressure occurs as the natural gas flows through a variable cross-sectional area. This pressure drop is inherently associated with a temperature change governed by the Joule–Thomson effect. Therefore, the thermodynamic response of the gas, particularly the temperature differential between upstream and downstream of the valve, is directly linked to the valve’s operating condition and flow characteristics^11^.

Energy demand is anticipated to persistently rise in the forthcoming years to satisfy the requirements of an expanding human population^12–14^. The impetus for process automation is driven by escalating labour costs and heightened quality demands, rendering the monitoring of production systems imperative^15,16^. From a condition monitoring perspective, this relationship provides an opportunity to define a physics-based diagnostic parameter, where deviations in the expected thermodynamic behaviour may indicate abnormalities such as valve degradation, improper flow regulation, or performance loss. As a result, comprehensive research has been undertaken globally in the domain of tool and process condition monitoring, which has been a primary research focus for over two decades and is essential in contemporary industrial systems^17^.

Existing condition monitoring techniques for control valves include flow-based, pressure-based, thermal imaging, and vibration-based methods^18^. Flow and pressure measurements are commonly used due to their availability in industrial systems; however, they may not always be sensitive to changes in valve condition. Vibration-based techniques can provide early fault detection but often require additional sensors and may be affected by environmental noise and structural interactions. Parameters for condition monitoring in industrial equipment must be meticulously chosen to detect critical indicators of potential faults in the machinery^19,20^. An equation of state (EOS) characterises the thermodynamic state of a fluid combination and its vapour-liquid phase equilibrium behaviour. An optimal equation of state should precisely forecast the thermodynamic properties of any fluid across an extensive spectrum of pressure, temperature, and composition for both vapour and liquid phases^21,22^. The AGA8-92DC EOS, introduced by the American Gas Association in 1992 and subsequently published in ISO12213-2, remains the industry standard for accurately predicting the density or compressibility factor of natural gas. Additional correlation equations were published, including GB/T 17747.2–2011 from China and GERG-2008 produced by the Groupe Européen de Recherches, which is endorsed by ISO 20765-2. Nevertheless, AGA8-92DC is extensively employed to assess the precision of alternative correlations and serves as a source for data training and testing^23,24^. The AGA8-92DC equation of state was selected due to its balance between computational efficiency and accuracy within the operating range considered in this study. While more detailed formulations such as AGA8-Detail can provide higher accuracy, particularly for complex gas compositions, the wide adoption of AGA8-92DC makes it well-suited for real-time condition monitoring in industrial applications^25,26^. Moreover, the pressure and temperature ranges investigated fall within the domain where AGA8-92DC provides reliable predictions. Hence, this study employs the AGA8-92DC method to compute temperature differentials between the upstream and the downstream sides of a gas turbine fuel control valve with a variable cross-section, analysing two fluid mixtures: natural gas and pure methane, at two distinct inlet pressures.

While previous studies on control valve condition monitoring have primarily focused on vibration signals, flow characteristics, or data-driven artificial intelligence approaches, few studies considered thermodynamically derived indicators linked to valve geometry and gas expansion behaviour. The key contribution of this work is introducing a novel monitoring parameter based on the temperature differential between the upstream and the downstream flow as a function of valve cross-sectional area variation, enabling accurate prediction of gas behaviour. Additionally, the temperature gradient is proposed as a diagnostic feature for condition monitoring, offering a new perspective that complements existing data-driven and signal-based approaches. The proposed approach utilises a thermodynamic indicator derived from the Joule–Thomson effect, linking temperature variation directly to valve operation. This provides a complementary perspective that reflects the physical behaviour of the working fluid and can enhance monitoring capability when compared to conventional methods.

## Methods

In Joule-Thomson expansion, the temperature of a fluid either diminishes or increases based on its initial pressure and temperature. This phenomenon induces temperature variations in fluids during expansion.

The Joule-Thomson (JT) coefficient is defined as the temperature derivative with respect to pressure given at constant enthalpy, as expressed in Eq. (1):

1 $$\:{\mu\:}_{JT}={\left(\frac{\partial\:T}{\partial\:P}\right)}_{h}$$

Where $\:{\mu\:}_{JT}$ the JT coefficient, *P* is pressure, *T* is temperature, and *h* is Enthalpy. JT may be positive or negative according to state conditions. Positive values of the Joule-Thomson coefficient indicate cooling of the fluid stream as it passes through the isenthalpic throttle. The curve that connects all state points where the JT coefficient equals zero is known as the JT inversion curve. A key industrial application of the JT inversion curve is in the gas liquefaction processes. Due to the JT effect, the fluid state before throttling must lie close to the inversion curve. Specifically, the working fluid temperature must be less than the maximum inversion temperature to achieve the greatest temperature drop resulting from the decrease in pressure. It should be noted that the assumption of stable downstream pressure is valid primarily under steady-state or quasi-steady operating conditions. Henceforth, the proposed model can be interpreted as a steady-state thermodynamic baseline, against which transient deviations may be detected but not explicitly modelled.

The Joule-Thomson equation is derived using Maxwell’s equations for the fundamental thermodynamic relations^27^.

2 $$\:dh=Tds+vdp$$

Dividing Eq. (1) by $\:dp$ at constant $\:T$, the result is shown in Eqs. (3) and (4):

3 $$\:{\left(\frac{\partial\:h}{\partial\:P}\right)}_{T}=T{\left(\frac{\partial\:s}{\partial\:P}\right)}_{T}+v$$

4 $$\:{\left(\frac{\partial\:v}{\partial\:T}\right)}_{T}={-\left(\frac{\partial\:s}{\partial\:P}\right)}_{T}$$

Equation (2) is transformed by replacing Eq. (3), which is given by Eq. (5):

5 $$\:{\left(\frac{\partial\:h}{\partial\:P}\right)}_{T}=-T{\left(\frac{\partial\:v}{\partial\:T}\right)}_{P}+v$$

By considering a variable (thermodynamic property) that is a continuous function for Enthalpy, Eq. (6) is developed:

6 $$\:dh={\left(\frac{\partial\:h}{\partial\:T}\right)}_{p}dp+{\left(\frac{\partial\:h}{\partial\:p}\right)}_{T}dT$$

For constant heat capacity, Eq. (7) is used:

7 $$\:{C}_{p}={\left(\frac{\partial\:h}{\partial\:p}\right)}_{T}$$

By substituting Eqs. (5) and (7) into Eq. (6), Eq. (8) is derived:

8 $$\:dh={C}_{p}dT+\left[v-T{\left(\frac{\partial\:v}{\partial\:T}\right)}_{P}\right]dp$$

In the isenthalpic process, with $\:dh=0$; Eq. (7) is converted to Eq. (8) as shown by Eq. (9)

9 $$\:{\left( {\frac{{\partial \:T}}{{\partial \:P}}} \right)_h} = \frac{1}{{{C_p}}}[T{(\frac{{\partial \:v}}{{\partial \:T}})_P} - v]$$

After substituting $\:V=ZRT$, Eq. (10) is obtained^15^.

10 $$\:{\mu\:}_{J}=\frac{R{T}^{2}}{P\:{C}_{p}}{\left(\frac{\partial\:Z}{\partial\:T}\right)}_{P}$$

According to the provisions of AGA8/1992 and ISO-12213-2/1997^28,29^, the EOS for the compression factor of natural gas is given by Eq. (11):

11 $$\:Z=1+\frac{DB}{{K}^{3}}-D\sum\:_{n=13}^{18}{C}_{n}^{\mathrm{*}}\:{T}^{-{u}_{n}}+\sum\:_{n=13}^{58}{C}_{n}^{\mathrm{*}}\:{T}^{-{u}_{n}}\left({b}_{n}-{c}_{n}{k}_{n}{D}^{{k}_{n}}\right){D}^{{b}_{n}}{e}^{-{c}_{n}{D}^{{k}_{n}}}$$

Where D is the reduced density, B is the second virial coefficient, and $\:{C}_{n}^{*}$ is the temperature coefficient.

The reduced density related to molar density is given by Eq. (12):

12 $$\:D={K}^{3}\rho$$

Where $\:K\:\mathrm{a}\mathrm{n}\mathrm{d}\:\rho\:$ are the mixture size parameter and density, respectively.

Coefficients $\:G,U,Q,F$ are the mixture orientation parameter, the mixture energy parameter, the quadrupole parameter, and the mixture high-temperature parameter, respectively.

The mixture size parameter can be calculated by using Eq. (13):

13 $$\:{K^5} = {\left( {\sum {\:_{i = 1}^N} {x_i}{K_i}^{5/2}} \right)^2} + 2\sum {\:_{i = 1}^{N - 1}} \sum {\:_{j = i + 1}^N} {x_i}{x_j}({K_{ij}}^5 - 1){({K_i}{K_j})^{\frac{5}{2}}}\:$$

Where $\:{K}_{ij}\:\mathrm{a}\mathrm{n}\mathrm{d}\:{K}_{i}$ are the binary interaction parameter and the corresponding size parameters.

The mixture orientation parameter can be calculated by using Eqs. (14) and (15):

14 $$\:G=\sum\:_{i=1}^{N}{x}_{i}{G}_{i}+2\sum\:_{i=1}^{N-1}\sum\:_{j=i+1}^{N}{x}_{i}{x}_{j}\left({G}_{ij}^{\mathrm{*}}-1\right)\left({G}_{i}+{G}_{j}\right)$$

That:

15 $$\:{G}_{ij}=\frac{{{G}_{ij}}^{\mathrm{*}}\left({G}_{i}+{G}_{j}\right)}{2}$$

Where $\:{{G}_{ij}}^{*}\:\mathrm{a}\mathrm{n}\mathrm{d}\:\:{G}_{i}\:$ are the corresponding binary interaction parameters and orientation parameters for the i-th component, respectively.

The mixture energy parameter can be calculated by using Eqs. (16) and (17)

16 $$\:{U^5} = {\left( {\sum {\:_{i = 1}^N} {x_i}{E_i}^{5/2}} \right)^2} + 2\sum {\:_{i = 1}^{N - 1}} \sum {\:_{j = i + 1}^N} {x_i}{x_j}({U_{ij}}^5 - 1){({E_i}{E_j})^{\frac{5}{2}}}$$.

That:

17 $$\:{{E}_{ij}}^{.}={{E}_{ij}}^{\mathrm{*}}\left({E}_{i}{E}_{j}\right)$$

Where $\:{{E}_{ij}}^{*},\:{\:E}_{i}\:,{U}_{ij}$ are the corresponding binary interaction parameters, the characteristic energy parameter K for the i-th component, and the binary interaction parameter for mixture energy, respectively.

The Quadrupole parameter is defined by Eq. (18)

18 $$\:Q={\sum\:_{i=1}^{N}{x}_{i}{Q}_{i}}^{1}$$

Where $\:{Q}_{i}$ is the Quadrupole parameter for the i-th component.

The mixture high-temperature parameter can be calculated by using Eq. (19)

19 $$\:F={\sum\:_{i=1}^{N}{{x}_{i}}^{2}{F}_{i}}^{.}$$

Where $\:{F}_{i}$ is the high-temperature parameter for the i-th component.

The gas mixture molar mass $\:{M}_{r}$ is calculated from the composition using Eq. (20):

20 $$\:{M}_{r}=\sum\:_{i=1\:}^{N}{x}_{i}{M}_{ri}$$

Where $\:{M}_{r},$ $\:{x}_{i}{\:,and\:M}_{ri\:}\:\:$are the molecular weight, mole fraction, and molar mass of the ith component mix, respectively.

The Second virial Coefficient is defined by Eq. (21):

21 $$\:B=\sum\:_{n=1}^{18}{a}_{n}{T}^{-{u}_{n}}\times\:\sum\:_{i=1}^{N}\sum\:_{j=1}^{N}{x}_{i}{x}_{j}{B}_{nij}^{\mathrm{*}}{{E}_{ij}}^{{u}_{n}}{{(K}_{i}{K}_{j})}^{\frac{3}{2}}$$

Where $\:{B}_{nij}^{*}\:and\:{E}_{ij}$ are defined according to Eq. (22):

22 $$\:{B}_{nij}^{\mathrm{*}}={{{(G}_{ij}}^{.}+1-{g}_{n})}^{{g}_{n}}\times\:{\left({Q}_{i}{Q}_{j}+1-{q}_{n}\right)}^{{q}_{n}}{\left({F}_{i}^{\frac{1}{2}}{F}_{j}^{\frac{1}{2}}+1-{f}_{n}\right)}^{{f}_{n}}{\times\:\left({S}_{i}^{.}{S}_{j}^{.}+1-{s}_{n}\right)}^{{s}_{n}}{\left({W}_{i}^{.}{W}_{j}^{.}+1-{w}_{n}\right)}^{{w}_{n}}\:$$

Where {$\:{a}_{n}\},\:{\{f}_{n}\},\:{\{g}_{n}\},{\{q}_{n}\},\:{\{s}_{n}\},\:{\{w}_{n\:}\}\:\mathrm{a}\mathrm{n}\mathrm{d}\:\left\{{u}_{n}\right\}\:$are the equations of parameters.

$\:\left\{{E}_{i}\right\},\:\:\left\{{F}_{i}\right\},\:\:\left\{{G}_{i}\right\},\:\:\left\{{K}_{i}\right\},\:\:\left\{{Q}_{i}\right\},\:\:\left\{{S}_{i}\right\}\:\:\mathrm{a}\mathrm{n}\mathrm{d}\:\:\left\{{W}_{i}\right\}$ are the corresponding characterisation parameters. The temperature-dependent coefficients $\:\:\:\{{C}_{n}^{*};n=\mathrm{1,2},\dots\:,58\}$ are defined by Eq. (23):

23 $$\:{C}_{n}^{\mathrm{*}}={a}_{n}{\left(G+1-{g}_{n}\right)}^{{g}_{n}}{\left({Q}^{2}+1-{q}_{n}\right)}^{{q}_{n}}{\left(F+1-{f}_{n}\right)}^{{f}_{n}}{U}^{{u}_{n}}{T}^{-{u}_{n}}$$

The partial derivative of pressure to temperature at constant molar volume, and the partial derivative of molar volume to temperature at constant pressure, are given by Eqs. (24)–(37):

24 $$\:{\left(\frac{\partial\:Z}{\partial\:T}\right)}_{p}=\frac{R{\left(TZ\right)}^{2}{C}_{3}-PZ[T{K}^{3}{C}_{0}+{C}_{4}]}{R{\left(TZ\right)}^{2}+PT{C}_{4}}$$

For the first derivative virial coefficient,

25 $$\:{B}^{{\prime\:}}=-\sum\:_{n=1}^{18}{a}_{n}{{u}_{n}T}^{-{u}_{n}-1}\times\:\sum\:_{i=1}^{N}\sum\:_{j=1}^{N}{x}_{i}{x}_{j}{B}_{nij}^{\mathrm{*}}{{E}_{ij}}^{{u}_{n}}{{(K}_{i}{K}_{j})}^{\frac{3}{2}}$$

Where the coefficients $\:{C}_{3},\:{C}_{0},\:{C}_{4}\:\:$are defined as:

26 $${C_3} = \sum {\:_{n = 13}^{58}} (C_n^{ * '}D_n^ * )\:$$

27 $$\:{C}_{0}=\sum\:_{n=13}^{18}{{C}_{n}^{\mathrm{*}}}^{{\prime\:}}-\frac{{B}^{{\prime\:}}}{{K}^{3}}$$

Where $\:{{C}_{n}^{*}}^{{\prime\:}}$is defined as:

28 $$\:{{C}_{n}^{\mathrm{*}}}^{{\prime\:}}=\frac{-{u}_{n}}{T}{{C}_{n}^{\mathrm{*}}}^{.}$$

and

29 $$\:{{C}_{n}^{\mathrm{*}}}^{{\prime\:}{\prime\:}}=\frac{-{u}_{n}+1}{T}{{C}_{n}^{\mathrm{*}}}^{{\prime\:}}$$.

For compute $\:{D}_{n}^{*}\:$ using below formulation:

30 $$\:{D}_{n}^{\mathrm{*}}=\left({b}_{n}-{c}_{n}{k}_{n}{{\rho\:}_{r}}^{{k}_{n}}\right){{\rho\:}_{r}}^{{b}_{n}}{e}^{-{c}_{n}{{\rho\:}_{r}}^{{k}_{n}}}$$

31 $$\:{C}_{4}={C}_{5}+\sum\:_{n=13}^{58}\left({C}_{n}^{\mathrm{*}}{D}_{n}^{1}\right)$$

Where is $$\:{C}_{5}$$:

32 $$\:{C}_{5}=B-{K}^{3}\sum\:_{n=13}^{18}{(C}_{n}^{\mathrm{*}})$$

For the first and second derivative $\:{C}_{5}$ respectively:

33 $${C_5}^{\prime \:} = B\prime \: - {K^3}\sum {\:_{n = 13}^{18}} (C_n^{ * '})$$

and

34 $${C_5}^{\prime \:\prime \:} = B\prime \:\prime \: - {K^3}\sum {\:_{n = 13}^{18}} (C_n^{ * ''})$$

Where

35 $$\:D_n^1 = {K^3}\left[ {b_n^2 - {c_n}{k_n}\left( {2{b_n} + {k_n} - {c_n}{k_n}\rho {\:_r}^{{k_n}}} \right)\rho {\:_r}^{{k_n}}} \right]\: \times \:\rho {\:_r}^{{b_n} - 1}{e^{ - {c_n}\rho {\:_r}^{{k_n}}}}$$.

And

36 $$\:{B}^{{\prime\:}{\prime\:}}=\sum\:_{n=1}^{18}{a}_{n}{{u}_{n}({u}_{n}+1)T}^{-{u}_{n}-2}\times\:\sum\:_{i=1}^{N}\sum\:_{j=1}^{N}{x}_{i}{x}_{j}{B}_{nij}^{\mathrm{*}}{{E}_{ij}}^{{u}_{n}}{{(K}_{i}{K}_{j})}^{\frac{3}{2}}$$

37 $$\:{D}_{n}^{2}={K}^{3}\{{D}_{n}^{1}\left[\left({b}_{n}-1\right){{\rho\:}_{r}}^{-1}\right]\:\:\:\:\:\:+{k}_{n}{{\rho\:}_{r}}^{-1}\left(1-{c}_{n}{{\rho\:}_{r}}^{{k}_{n}}\right)+{K}^{3}{k}_{n}\left({{c}_{n}}^{2}{{k}_{n}}^{2}{{\rho\:}_{r}}^{2{k}_{n}}-{b}_{n}^{2}\right){{\rho\:}_{r}}^{{b}_{n}-2}{e}^{-{c}_{n}{{\rho\:}_{r}}^{{k}_{n}}}\}$$.

To calculate $\:{C}_{p}$, the molar heat capacity can be defined by^30^ as shown by Eq. (38):

38 $$\:{C}_{p}={C}_{pI}-RT\left(T{\phi\:}^{{\prime\:}{\prime\:}}+2{\phi\:}^{{\prime\:}}\right)$$

Where $\:{\phi\:}^{{\prime\:}{\prime\:}}\:\mathrm{a}\mathrm{n}\mathrm{d}\:{\phi\:}^{{\prime\:}}\:are\:the\:$first and second derivatives of the gas fugacity coefficient,

$\:R\:\mathrm{a}\mathrm{n}\mathrm{d}\:{C}_{pI}$ are constant gas molar and Ideal molar heat capacity respectively.

Fugacity coefficients are defined by^31^ as shown by Eq. (39):

39 $$\:\phi\:={\int\:}_{0}^{p}\left(Z-1\right)\frac{dp}{p}=Z-1-\mathrm{ln}Z+{C}_{5}{\rho\:}_{m}+\sum\:_{N=13}^{58}{C}_{n}^{\mathrm{*}}{\rho\:}_{r}^{{b}_{n}}{e}^{-{c}_{n}{\rho\:}_{r}^{{b}_{n}}}$$

The first and second derivatives of the coefficient of gas fugacity are given by Eqs. (40)–(48):

40 $$\:{\phi\:}^{{\prime\:}}={Z}^{{\prime\:}}\left(1-\frac{1}{Z}\right)+{{C}_{5}}^{{\prime\:}}{\rho\:}_{m}+{C}_{5}{\rho\:}_{m}^{{\prime\:}}+\sum\:_{N=13}^{58}{{C}_{n}^{\mathrm{*}}}^{{\prime\:}}{\rho\:}_{r}^{{b}_{n}}{e}^{-{c}_{n}{\rho\:}_{r}^{{k}_{n}}}+\frac{{\rho\:}_{r}}{{{\rho\:}_{r}}^{{\prime\:}}}\sum\:_{N=13}^{58}{C}_{n}^{\mathrm{*}}{D}_{n}^{\mathrm{*}}$$

The second derivative of the coefficient of gas fugacity is defined by:

41 $$\begin{aligned} & \:{\phi\:}^{{\prime\:}{\prime\:}}={Z}^{{\prime\:}{\prime\:}}\left(1-\frac{1}{Z}\right) +\frac{{{Z}^{{\prime\:}}}^{2}}{{Z}^{2}}+{{C}{5}}^{{\prime\:}}{{\rho\:}{m}}^{{\prime\:}}+{\rho\:}{m}{{C}{5}}^{{\prime\:}{\prime\:}}+{C}{5}{{\rho\:}{m}}^{{\prime\:}{\prime\:}} \\& +\sum\:{N=13}^{58}{{C}{n}^{\text{}}}^{{\prime\:}{\prime\:}}{\rho\:}{r}^{{b}{n}}{e}^{-{c}{n}{\rho\:}{r}^{{k}{n}}}+2{{\rho\:}{r}}^{{\prime\:}}{{\rho\:}{r}}^{-1} \\& \sum\:{N=13}^{58}{{C}{n}^{\text{}}}^{{\prime\:}}{D}{n}^{\text{}}+{{\rho\:}{r}}^{-2}\left({{\rho\:}{r}}^{{\prime\:}{\prime\:}}{\rho\:}{r}-{{{\rho\:}{r}}^{{\prime\:}}}^{2}\right)\\& \sum\:{N=13}^{58}{C}{n}^{\text{}}{D}{n}^{\text{}} +{K}^{-3}{{{\rho\:}{r}}^{{\prime\:}}}^{2}{{\rho\:}{r}}^{-1}\sum\:{N=13}^{58}{C}{n}^{\text{}}{D}{n}^{1}\end{aligned}$$

Where:

42 $$\:{\rho\:}_{m}=\frac{P}{RTZ}$$

And for the first and second derivatives corresponding to Eq. (40) are:

43 $$\:{{\rho\:}_{m}}^{{\prime\:}}=-\frac{P}{R}\left(\frac{Z+T{Z}^{{\prime\:}}}{{\left(TZ\right)}^{2}}\right)$$

44 $$\:{{\rho\:}_{m}}^{{\prime\:}{\prime\:}}=\frac{P}{R}\times\:\frac{2{\left(Z+T{Z}^{{\prime\:}}\right)}^{2}-TZ\left(2{Z}^{{\prime\:}}+T{Z}^{{\prime\:}{\prime\:}}\right)}{{\left(TZ\right)}^{3}}\:$$

By using Eqs. (22), (41) and (42) we can define $\:{Z}^{{\prime\:}{\prime\:}}$ as:

$$\:{Z}^{{\prime\:}{\prime\:}}=\frac{1}{R{\left(TZ\right)}^{3}+P{T}^{2}Z{Z}_{1}}\times\:\left\{2P{Z}_{1}{[\left(Z+T{Z}^{{\prime\:}}\right)}^{2}+TZ{Z}^{{\prime\:}}\right]-2PTZ\left({{C}_{5}}^{{\prime\:}}+\sum\:_{N=13}^{58}{{C}_{n}^{\mathrm{*}}}^{{\prime\:}}{D}_{n}^{1}\right)\left(Z+T{Z}^{{\prime\:}}\right)$$

45 $$\: + \frac{{{{\left[ {P\left( {Z + T{Z^{\prime \:}}} \right)} \right]}^2}}}{{RTZ}}\sum {\:_{n = 13}^{58}} C_n^{\mathrm{*}}D_n^2 + P{\left( {TZ} \right)^2}{C_5}^{\prime \:\prime \:} + R{\left( {TZ} \right)^3} + \sum {\:_{N = 13}^{58}} C_n^{*''}D_n^*\}$$

Where $\:{Z}_{1}$ is defined as:

46 $$\:{Z}_{1}={C}_{5}+\sum\:_{n=13}^{18}{(C}_{n}^{\mathrm{*}}\:{D}_{n}^{1})$$

The ideal heat capacity can be calculated as:

47 $$\:{C}_{PI}=\sum\:_{i=1}^{N}{x}_{i}{C}_{p}^{i}$$

where $\:{x}_{i}$ is the molar fraction of component i and $\:{C}_{p}^{i}$ is the molar heat capacity of component i in the gas mixture.

The molar heat capacities of the optimal gas mixture components can be estimated by the DIPPR/AIChE generic equations:

48 $$\:{{C}_{p}^{i}={{a}_{j}+b}_{j}\left(\frac{{c}_{j}/T}{Sinh\left({c}_{j}/T\right)}\right)}^{2}+{d}_{j}{\left(\frac{{e}_{j}/T}{Cosh\left({e}_{j}/T\right)}\right)}^{2}$$

Where $\:{C}_{p}^{i}$ is the molar heat capacity of component j of the optimal gas mixture, for every value of component a, b, c, d, e given in Table 1.

**Table 1:** The DIPPR/AIChE ideal gas heat capacity constant.

Natural Gas	Dippr/AIChe ideal gas heat capacity constant				
	a	b	C	d	e
Methane	33,298	79,933	2086.9	41,602	991.96
Ethane	40,326	134,220	165.5	73,223	752.87
Propane	51,930	192,490	1626.5	116,800	723.6

The corresponding temperature change of the natural gas for the upstream and downstream is shown in Eq. (49)^32^:

49 $$\:({T}_{2\:}-{T}_{1\:})=\varDelta\:T={\mu\:}_{j}\varDelta\:\omega$$

Where$\:\:{T}_{1\:}\:\mathrm{a}\mathrm{n}\mathrm{d}\:\:{T}_{2\:}$ are the upstream and downstream temperatures.

The pressure loss across an area can be defined by Eq. (50)^33^:

50 $$\:\varDelta\:\omega\:=\frac{\sqrt{1-{\left(\xi\:\right)}^{4}\left(1-{C}^{2}\right)}-C{\left(\xi\:\right)}^{2}}{\sqrt{1-{\left(\xi\:\right)}^{4}\left(1-{C}^{2}\right)}+C{\left(\xi\:\right)}^{2}}\varDelta\:p$$

**Fig. 3:**
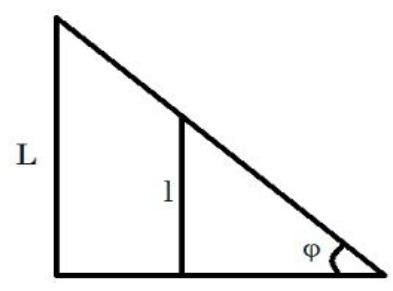
Schematic of variation cross area with angle 0–90°.

To model variations within the cross-sectional area and the associated temperature differential across the control valve, a variable plate, illustrated in Fig. 3, was considered. Modulating the plate angle between 0° and 90° enables precise control of the effective flow area. Equation (50) and Fig. 3, $\:C$ indicates the coefficient of discharge, while $\:\xi\:$ is the area variation coefficient ($\:0<\xi\:$*< L*); $\:\xi\:=L\:sin\left(\phi\:\right)$.

## Results and discussion

**Fig. 4:**
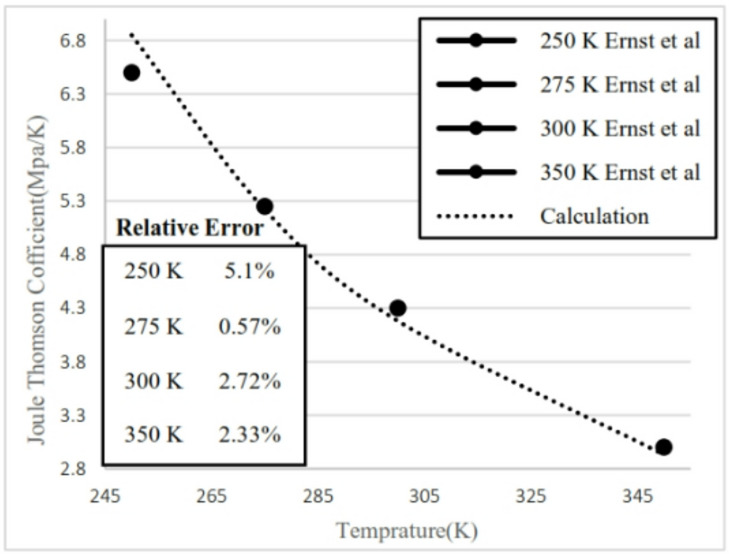
Validation Calculated Joule Thomson Coefficient and Ernst et al.^38^ Result in temperature (250, 275, 300 and 350 K) at pressure 5 MPa.

The implementation of measurement standards and calculation algorithms through object-oriented programming facilitates their seamless integration into the measurement system. Classes govern items as primary entities, whereas inheritance structures classes into hierarchies, enhancing their management efficiency^34,35^. The software object’s interface does calculations for the Joule-Thomson effect and natural gas properties in compliance with ISO 12213-2^22^. AGA8-92DC has been utilised to compute the output parameter^36^. The computation method for natural gas density, compressibility factor, molar heat capacity, downstream temperature, and the Joule–Thomson coefficient has been executed utilising a Visual Basic 16.9 for Applications (VBA) program, adhering to an object-oriented programming paradigm^37^. The input parameters consist of pressure, temperature, and molar percentages of natural gas constituents. The output parameters include density, compressibility factor, molar heat capacity, Joule–Thomson coefficient, and the temperature differential between downstream and upstream. To corroborate the theoretical calculations, experimental data from^38^ were employed, utilising the natural gas mixture specified in Table 2 for the Joule–Thomson coefficient parameter. The assessment of the Joule–Thomson coefficient at a pressure of 5 MPa was conducted by juxtaposing theoretical outcomes across a temperature spectrum of 245–345 K with four temperature data points obtained from experimental observations. The relative discrepancy between the experimental data and the estimated values for the four temperature locations is illustrated in Fig. 4. The presented results are obtained from a deterministic thermodynamic model; therefore, conventional statistical error bars based on repeated measurements are not directly applicable.

**Table 2:** Identical natural gas mixtures with mole fractions.

Component	Mole fraction
Methane	0.79942
Ethane	0.05029
Propane	0.03000
Nitrogen	0.09939
Carbon Dioxide	0.02090

**Fig. 5:**
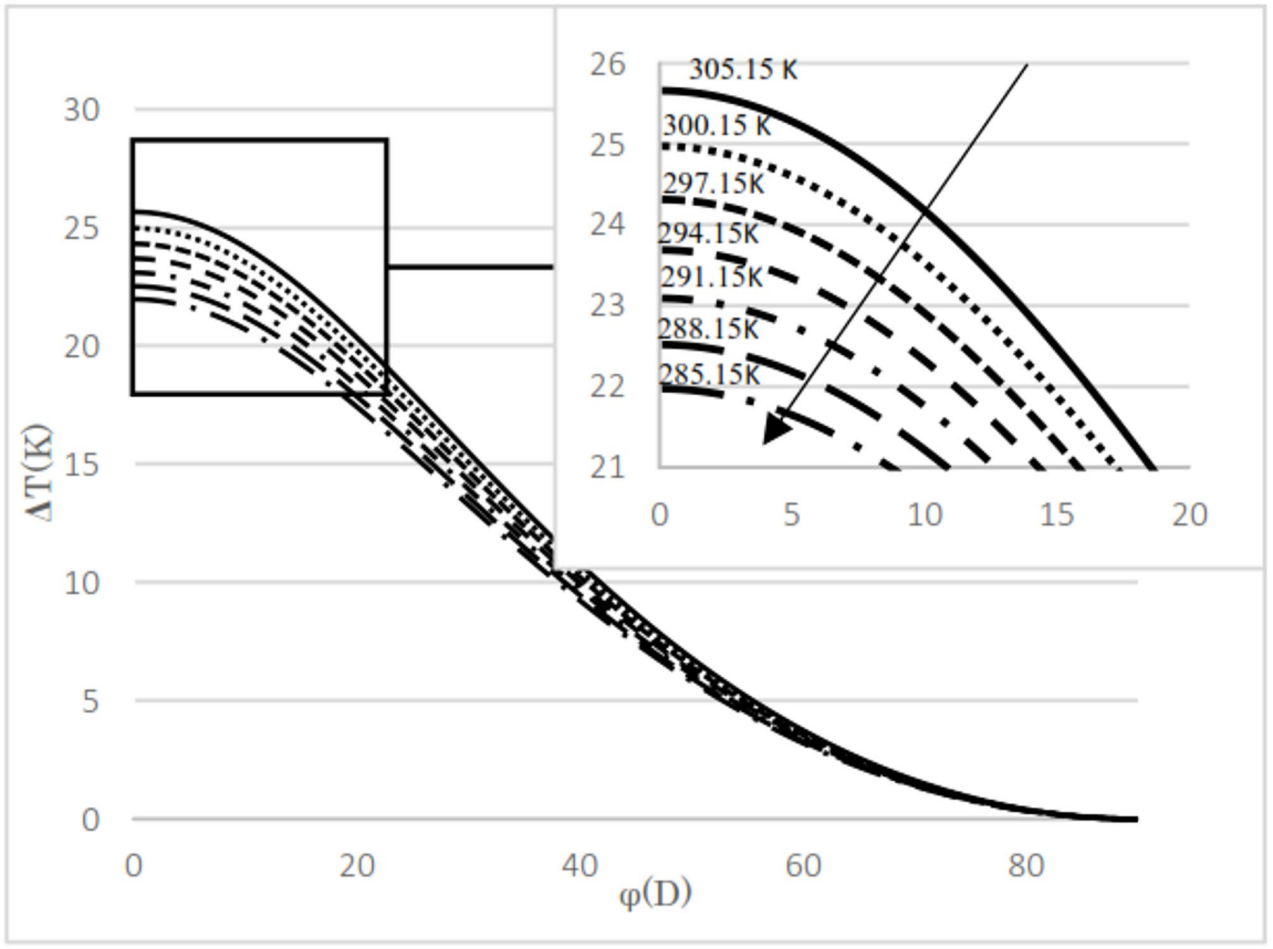
Temperature difference between upstream and downstream with angle at 5.4 MPa using natural gas component 1.

**Fig. 6:**
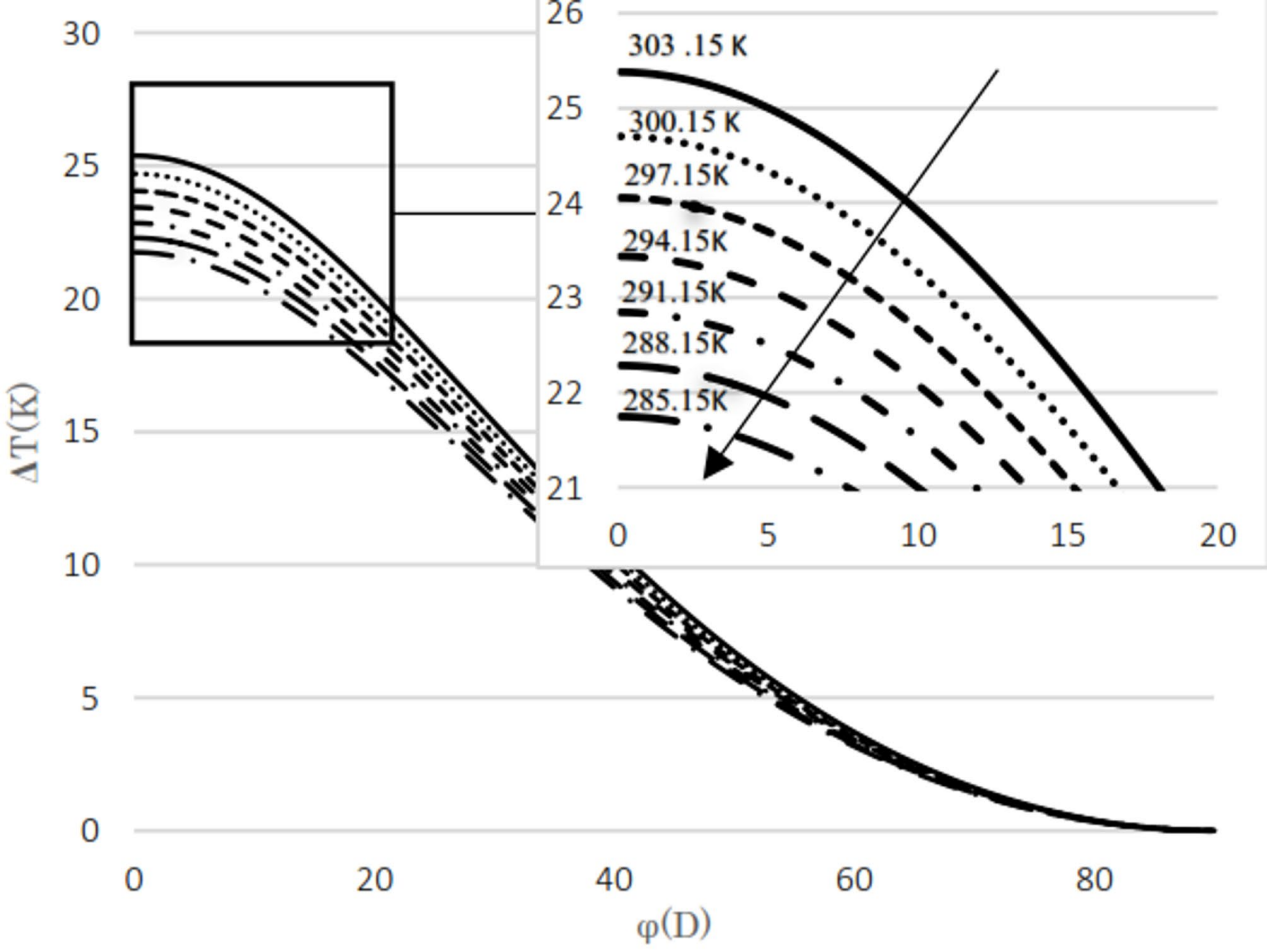
Temperature difference between upstream and downstream with angle at 2 MPa using natural gas component 2.

**Fig. 7:**
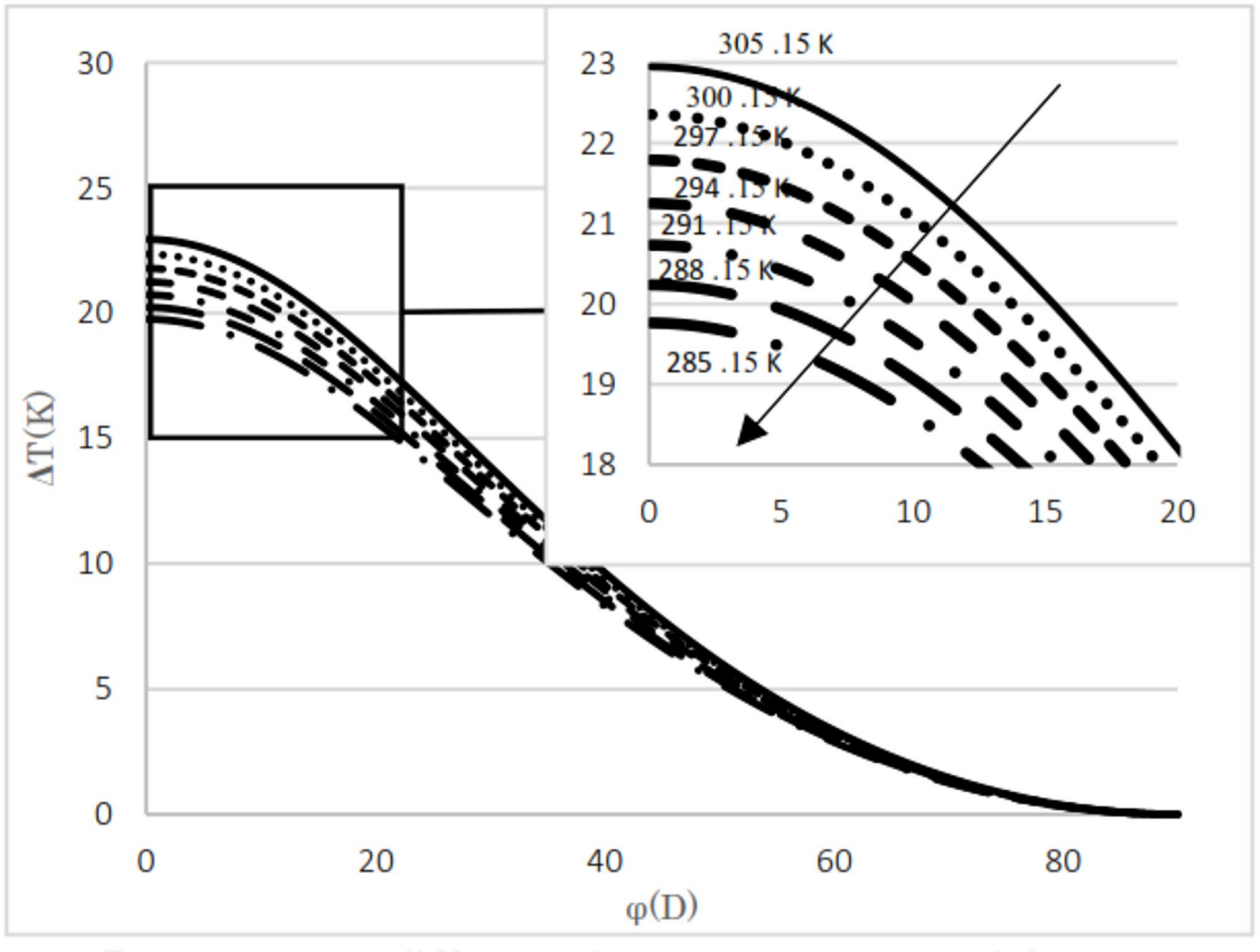
Temperature difference between upstream and downstream with angle at 5.4 MPa using pure methane.

**Fig. 8:**
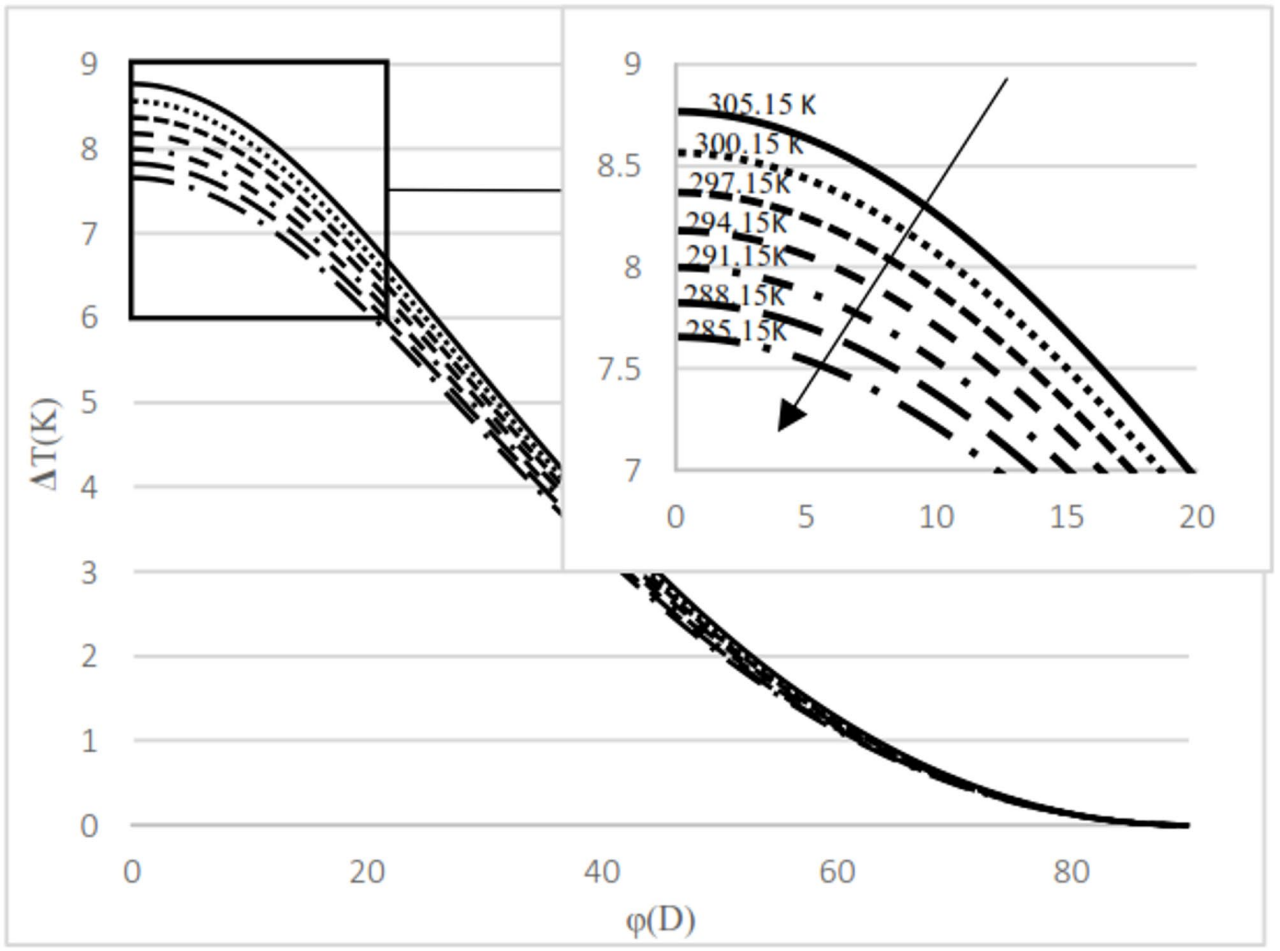
Temperature difference between upstream and downstream with angle at 2 MPa using natural gas component 2.

**Fig. 9:**
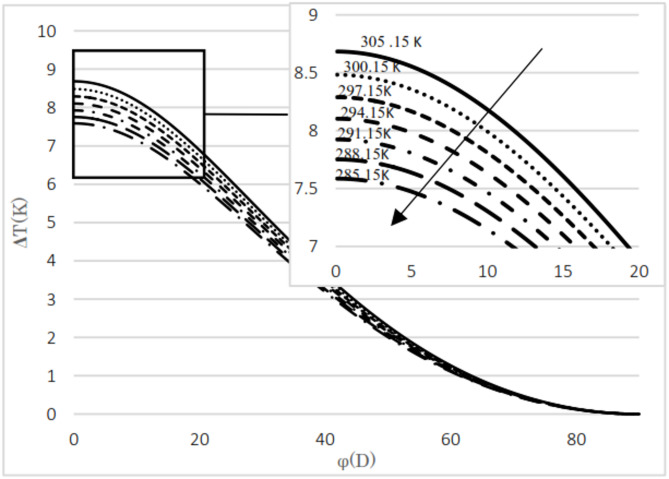
Temperature difference between upstream and downstream with angle at 5.4 MPa using pure methane.

**Fig. 10:**
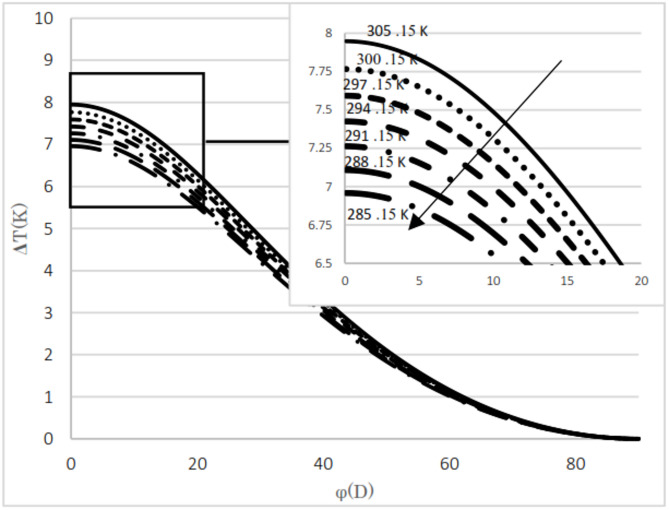
Temperature difference between upstream and downstream with angle at 2 MPa using pure methane.

The difference between the calculated and evaluated Joule–Thomson coefficients is shown in Fig. 4. The maximum absolute difference observed is 0.35, which increases as pressure decreases but remains below 5.1%. Additionally, at lower temperatures, the relative difference tends to increase. The calculated results are presented for two natural gas analyses as well as for pure methane. The input pressures considered are 5.4 MPa and 2 MPa, with the upstream temperatures ranging from 285 K to 303 K in 3 K increments. The mole fractions for the natural gas mixtures are listed in Table 3. The thermal variation between the upstream and the downstream for flowing natural gas and pure methane was investigated with respect to changes in cross-sectional area, as illustrated in Fig. 3. The temperature difference between upstream and downstream, along with the temperature gradient relative to the valve cross-sectional area, was calculated and presented. The temperature difference between the upstream and the downstream was calculated for two natural gas compositions and pure methane at a pressure gradient of 5.4 MPa, as shown in Figs. 5 and 6, and 7. Corresponding results for a pressure gradient of 2 MPa are presented in Figs. 8 and 9, and 10, respectively.

**Table 3:** Mole fractions of samples 1 and 2.

Component	Mole of Fraction (Sample 1)	Mole of Fraction (Sample 2)
Methane	90.8	91.2
Ethane	3.5	3
Propane	1.43	1.06
Iso butane	0.22	0.29
Normal butane	0.32	0.4
Normal pentane	0.01	0.05
Iso pentane	0.01	0.07
Nitrogen	3.1	3.5
Carbon dioxide	0.6	0.4
Normal hexane	0.01	0.02
Normal heptane	0	0.01

**Fig. 11:**
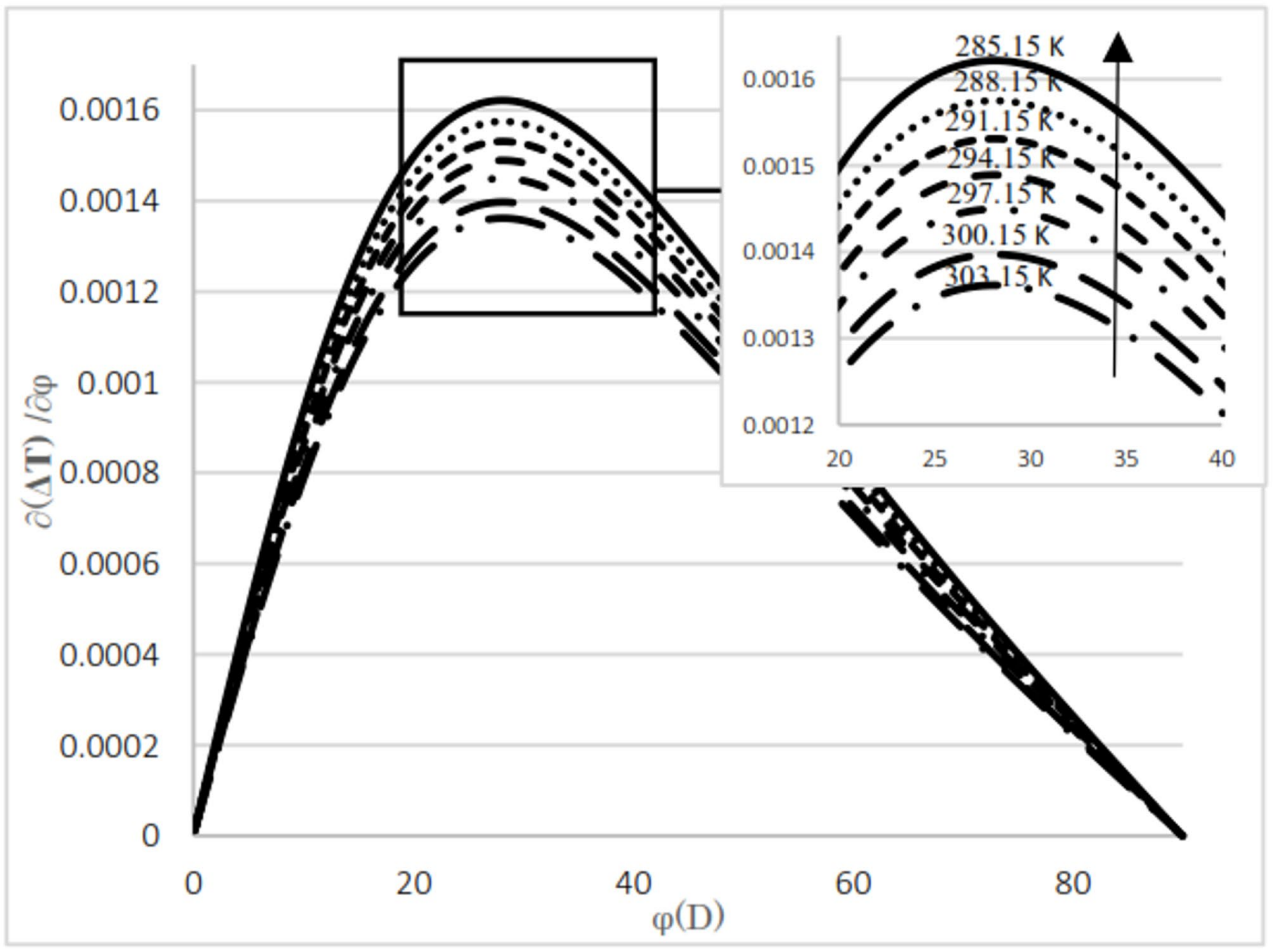
Difference temperature Gradient with angle at pressure 2 Mpa by using Pure methane.

**Fig. 12:**
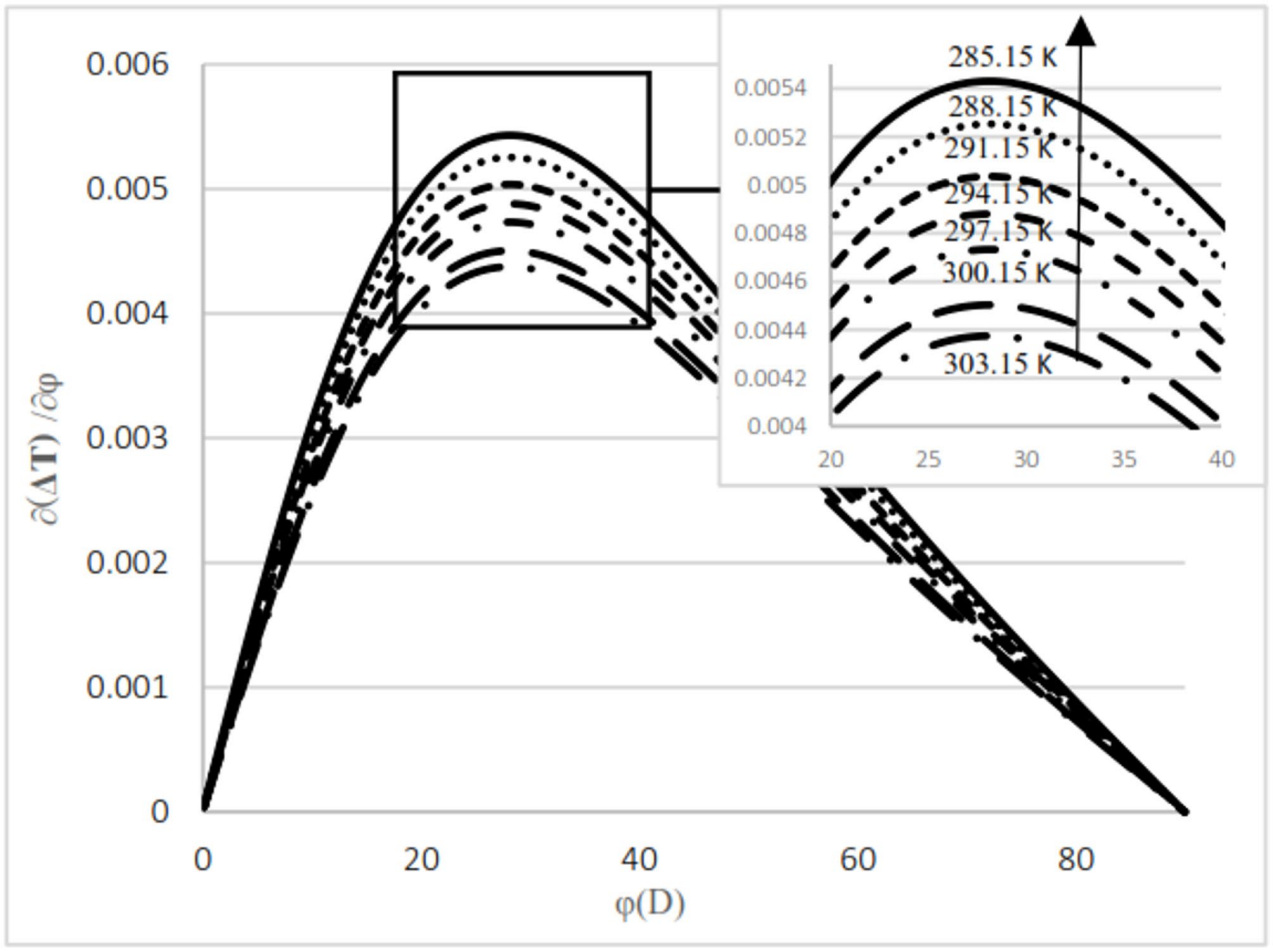
Difference temperature Gradient with angle at pressure 5.4 Mpa by using Pure methane.

As observed in both natural gas compositions and pure methane, at the same cross-sectional area, pressure gradient, and composition, the temperature difference between the upstream and the downstream is directly proportional to the inlet fluid temperature. According to the curves for gas compositions and pure methane, a higher percentage of chain alkanes, such as methane, leads to a reduced temperature difference between downstream and upstream at the same inlet fluid temperature, cross-sectional area, and pressure gradient. This effect is particularly evident in the pure methane figure. Additionally, across all results, as the cross-sectional area increases, corresponding to the valve angle approaching 90˚, the temperature difference between upstream and downstream approaches zero. At constant pressure gradient, same cross area and the upstream temperature, changing the percentage of natural gas components will cause negligible changes in the difference between upstream and downstream fluid temperatures, provided that the percentage of methane component in natural gas does not change significantly. The difference temperature gradient to the valve cross-sectional area for pure methane is presented in Figs. 11 and 12, at pressure gradients of 5.4 MPa and 2 MPa, respectively. At a constant pressure gradient, an increase in input temperature results in a reduction in the temperature difference and temperature gradient to the valve cross-sectional area.

According to Figs. 11 and 12, the difference in temperature gradient to the valve cross-sectional area increases by an angle of up to 28°, after which it decreases until reaching 90°. Additionally, the gradient is notably higher between angles of 30° and 40° compared to other positions. The results indicate that, at the same upstream temperature and cross-sectional area, an increase in pressure gradient leads to a corresponding increase in both the magnitude of the temperature difference between the upstream and the downstream and the temperature gradient relative to the valve cross-sectional area.

Unlike purely data-driven condition monitoring approaches, the proposed model establishes a dynamic baseline derived from thermodynamic principles, which is continuously updated based on real-time operating conditions. This reduces susceptibility to model drift caused by non-stationary operating environments. However, on long-term industrial applications, factors such as sensor drift, gradual equipment ageing, and unmodelled nonlinearities may introduce deviations between predicted and actual behaviour^39^. To mitigate these effects, periodic recalibration and validation of the model using operational data are recommended. While the Joule–Thomson coefficient predictions were validated against published experimental data, the full condition monitoring framework was not experimentally validated under real gas turbine operating conditions or fault scenarios. Therefore, the proposed model should be considered as a foundational step toward a comprehensive condition monitoring system. Future work will involve experimental implementation and validation using real-time operational data to assess its performance in detecting valve degradation and faults. The proposed model can be interpreted as a feature engineering step for condition monitoring, providing physically meaningful inputs for future statistical or machine learning-based diagnostic systems.

The sensitivity of condition monitoring performance to threshold selection was considered to assess its potential impact. Conceptually, small threshold variations would have limited impact in regions where the temperature gradient is high (i.e., strong sensitivity to valve position), while greater sensitivity to threshold selection is expected near fully open conditions (~ 90°), where the temperature differential approaches zero. This aspect is particularly relevant when integrating the proposed model into real-time monitoring systems. To evaluate the responsiveness of the proposed thermodynamic indicator, a sensitivity analysis was conducted by introducing small variations in the valve cross-sectional area and observing the corresponding change in the upstream–downstream temperature differential (ΔT). Table 4 summarises the relationship between relative changes in valve cross-sectional area and the resulting variation in temperature differential under representative operating conditions.

**Table 4:** Relationship between changes in valve cross-sectional area and variation in temperature differential.

Relative change in valve area (%)	ΔT (K)	Change in ΔT (K)	Relative Change in ΔT (%)
−5%	11.8	−1.2	−9.2%
−2%	12.6	−0.4	−3.1%
0% (Baseline)	13.0	0.0	0.0%
+ 2%	13.5	+ 0.5	+ 3.8%
+ 5%	14.3	+ 1.3	+ 10.0%

Although the present study does not directly model emissions, a simplified estimation can be made based on the relationship between combustion temperature and NOx formation. In gas turbines, small variations in fuel flow can lead to flame temperature fluctuations which may significantly influence NOx production^40,41^. By improving control valve performance and reducing fuel flow instability, the proposed monitoring approach can help stabilise combustion conditions. Based on typical combustion sensitivity reported in the literature, this stabilisation may correspond to a potential NOx emission reduction of approximately 3–8% under normal operating conditions. This estimate is indicative and intended to demonstrate the potential environmental benefit of improved valve monitoring. A more accurate quantification would require coupling the present model with detailed combustion and emissions simulations, which will be addressed in future work. In this sense, the contribution of this work lies in feature construction for condition monitoring, rather than in the final diagnostic decision-making layer.

## Conclusions and future work

In this study, the AGA8-92DC method is used to calculate temperature differences between the upstream and downstream sides of a gas turbine fuel control valve with a changing cross-section, considering two fluid mixtures, natural gas and pure methane, at two different inlet pressures. The effect of the valve cross-sectional area on the temperature of the gas fluid at different inlet temperatures is analysed. In all cases, as the cross-sectional area increases, the downstream temperature tends to approach the upstream temperature. When the valve is fully open at a 90° angle, the upstream and downstream temperatures become equal. Variations in fluid temperature across the cross-section can be utilised for monitoring control valve conditions and verifying the correct operation of the natural gas injection valve in a gas turbine power plant, resulting in more accurate control of exhaust emissions. Under similar physical conditions, near-identical results are observed for the two natural gas composition samples, which simplifies condition monitoring.

From a practical perspective, the proposed model can be utilised for real-time monitoring of control valve performance, providing early indications of abnormal behaviour and supporting predictive maintenance strategies. Such strategy can contribute to improved combustion stability and operational efficiency with potential NOx emission reduction of approximately 3–8% under normal operating conditions.

Based on the results of this study, for effective condition monitoring of the control valve, it is necessary to select an appropriate range for the temperature difference between the upstream and the downstream. When the temperature gradient ratio across the cross-sectional area is significant, condition monitoring can be performed more accurately. While this enables the identification of deviations potentially associated with valve degradation, a key limitation of the current study is that it does not explicitly separate random measurement noise from degradation effects. Another technical limitation is that the accuracy of the model is dependent on the quality of input measurements which may also be influenced by sensor noise. Furthermore, external environmental factors, such as ambient temperature variations, are not explicitly considered in the current framework. These limitations highlight the need for future work focusing on transient modelling, uncertainty quantification, and integration with adaptive monitoring techniques to enhance robustness under real industrial operating conditions.

Future work will focus on integrating the proposed thermodynamic model with advanced statistical filtering and machine learning techniques for larger datasets to enable robust discrimination between noise and actual valve deterioration. Another future direction can focus on incorporating uncertainty quantification techniques, such as Monte Carlo simulation and probabilistic analysis to quantify the impact of threshold variation on detection performance and provide confidence intervals to further strengthen the statistical interpretation of the results and provide experimental validation.

## Data Availability

Data is included in the manuscript.
